# Comparative demography of two common scleractinian corals: *Orbicella annularis* and *Porites astreoides*

**DOI:** 10.7717/peerj.3906

**Published:** 2017-10-27

**Authors:** Francisco J. Soto-Santiago, Alex Mercado-Molina, Koralis Reyes-Maldonado, Yaileen Vélez, Claudia P. Ruiz-Díaz, Alberto Sabat

**Affiliations:** 1 Department of Environmental Sciences, University of Puerto Rico, Río Piedras, Puerto Rico; 2 Department of Biology, University of Puerto Rico, Río Piedras, Puerto Rico; 3 Sociedad Ambiente Marino Inc., San Juan, Puerto Rico; 4 Department of Biology, University of Puerto Rico, Bayamón, Puerto Rico

**Keywords:** Demography, Resilience, Caribbean, Scleractinians, Life table response analysis, Lambda, Akaike information criteria, Population ecology, *Porites astreoides*, *Orbicella annularis*

## Abstract

**Background:**

Studies directed at understanding the demography and population dynamics of corals are relatively scarce. This limits our understanding of both the dynamics of coral populations and our capacity to develop management and conservation initiatives directed at conserving such ecosystems.

**Methods:**

From 2012 to 2014, we collected data on the growth, survival, and recruitment rates of two common Caribbean coral species, the stress-tolerant *Orbicella annularis* and the weedy *Porites astreoides*. A set of size-based population matrix model was developed for two localities in Northeastern Puerto Rico and used to estimate population growth rates (λ) and determine the life cycle transition(s) that contribute the most to spatiotemporal differences in λs. The model was parameterized by following the fate of 100 colonies of each species at the two sites for two years.

**Results:**

Our data indicate that spatial variability in vital rates of both species was higher than temporal variability. During the first year, populations of *O. annularis* exhibited λs below equilibrium at Carlos Rosario (0.817) and Palomino (0.694), followed by a considerable decline at both sites during the second year (0.700 and 0.667). Populations of *P. astreoides* showed higher λs than *O. annularis* during the first census period at Carlos Rosario (0.898) and Palomino (0.894) with a decline at one of the sites (0.681 and 0.893) during the second census period. Colony fate in both species exhibited a significant interaction with respect to location but not to time (*G*2 = 20.96; *df* = 3 for *O. annularis* and *G*2 = 9.55; *df* = 3 for *P. astreoides*).

**Discussion:**

The similar variability of λs as well as the similar survival rates for both species during the two-year census period (2012–2014) show similar variability on demographic patterns in space and time. Our results suggest that location rather than time is important for the resiliency in coral colonies. Also, *P. astreoides* will show higher resistance to disturbance in the future than *O. annularis*.

## Introduction

Scleractinian corals are understood to be highly susceptible to environmental stress. However, species can vary widely in their capacity to withstand stressful environmental conditions such as high sea surface temperature, high sedimentation or epizootic events. Darling et al. (2012) categorized coral depending on their susceptibility to biological or physical disturbances. Paradoxically, in the classification proposed by Darling et al. (2012), *Orbicella annularis* (formerly known as *Montastraea annularis*) (Budd et al., 2012) is placed in the stress-tolerant category. There is evidence, however, indicating that the vital rates of this coral are sensitive to environmental stressors such as increased water temperature (Edmunds & Elahi, 2007; Hernández-Pacheco, Hernández-Delgado & Sabat, 2011) and is one of the most susceptible Caribbean corals to corallivorous snails (Potkamp, Vermeij & Hoeksema, 2017a, 2017b). It is, also, susceptible to coral diseases (Bruckner & Bruckner, 2006; Weil, Urreiztieta & Garzón-Ferreira, 2002; Weil, Cróquer & Urreiztieta, 2009) and is classified as “endangered” by the IUCN list (Carpenter et al., 2008). This apparent contradiction as to whether *O. annularis* is a “stress tolerant” species underlies our ignorance as to the biological basis of coral resilience to environmental stressors, and how to measure such resilience. On the other hand, *Porites astreoides* has been classified as a “weedy” coral species (Darling et al., 2012) and is considered as “least concern” by the IUCN list (Carpenter et al., 2008). This coral species is projected to increase its population size even when impacted with recurrent disturbance (Edmunds, 2010). Indeed, it is expected that coral cover in the Caribbean will be dominated by weedy species (Green, Edmunds & Carpenter, 2008). Based on the classification of Darling et al. (2012) we would expect similar demographic patterns on both species under similar conditions.

Documenting and understanding inter-species variability to environmental stress is key to predicting how corals will fare under the current and predicted climate change scenarios. For example, it has been suggested that coral reefs more resilient to climate change would be those dominated by coral species showing higher resistance to the effects of climate change rather than corals showing higher recovery from those effects (Coté & Darling, 2010). A powerful approach to evaluating the resistance of a species to environmental stress is by measuring the demographic or population level response of the species to various levels of environmental stress. Whether a coral population grows, declines or remains stable under a given environmental insult will depend on the effect of the environmental stressor on the colonies’ vital rates. In turn, vital rates are intimately related to the physiological state of corals (Anthony & Hoegh-Guldberg, 2003; Karim, Nakaema & Hidaka, 2015; Mayfield et al., 2012; McGinty, Pieczonka & Mydlarz, 2012). High temporal and spatial variability in vital rates should be indicative of a susceptible coral species, particularly if measured between sites or years that differ in the level of environmental stress to which colonies are subjected to. Resilient species, on the other hand, should exhibit little spatial and temporal variation in growth, survival or fecundity.

In this study, we examined the demography of *O. annularis* and *P. astreoides* from 2012 to 2014 at two localities in Northeastern Puerto Rico known to differ in benthic composition (cover). One of the reefs is dominated by non-calcareous algae, which is indicative of degraded conditions, whereas the other is characterized by a high coral cover which is characteristic of healthy coral reefs (Bruno et al., 2009; Done, 1992). The fact that both *O. annularis* and *P. astreoides* can be found inhabiting under these contrasting reefs conditions provides the opportunity to test whether this coral is indeed a stress-tolerant species. The working hypotheses are that if *O. annularis* is a stress-tolerant species, then its demographic behavior (1) should not vary considerably between localities, that is, we should observe no significant spatio-temporal difference in the vital rates and population growth rates; and (2) its demography would be comparable to that of a weedy species, *P. astreoides*. To test these hypotheses, we constructed and parameterized four size-based population matrices, one for each species, site and year. The matrices were used to (1) determine whether the studied populations were stable (λ = 1), increasing (λ > 1), or decreasing (λ < 1); (2) identify the life cycle transition(s) that accounted for the observed spatiotemporal differences in λ by performing life table response analyses; and (3) identify whether time or location have more effect on the fate of the colonies by using log-linear analyses. This study is the first to use demographic modeling to compare the population dynamics of *O. annularis* and *P. astreoides* in two contrasting coral reefs (regarding coral cover) to address how local stressors can affect their vital rates.

## Materials and Methods

**Figure 1:**
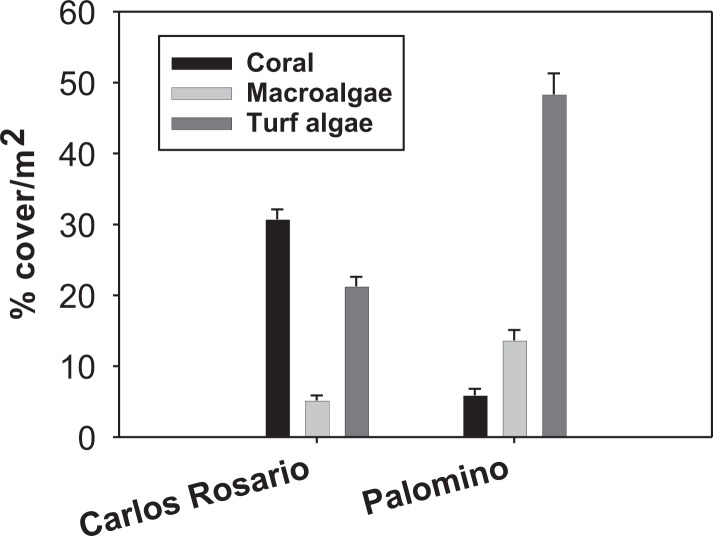
Reef benthic structure of both study sites.

Field work was conducted at two sites within the Puerto Rico archipelago. Carlos Rosario Reef (18°19′29″N, 65°19′60″W) is located within the Canal Luis Peña No-Take Natural Reserve on the island municipality of Culebra, approximately 30 km east of the Puerto Rico mainland. This reef is a linear fringing reef covering an area of approximately 30 ha with depths ranging from sea level to 14 m. Palomino (18°21′3″N, 65°33′58″W) is located within the Arrecifes de la Cordillera Natural Reserve, 6 km east from the coastal municipality of Fajardo on the Puerto Rico mainland. Carlos Rosario reef has a northwest orientation and experiences low to moderate wave action and long-period swells during the winter. Palomino reef has a southwest orientation and leeward from Palomino Island. Thus, it experiences less wave action. The reefs show a contrasting biological structure with a significantly higher coral cover (*F*(3, 159) = 83.90; *P* = 0.01) in Carlos Rosario Reef (Fig. 1) as studied on September 2012. Based on these results, and because high coral cover is characteristic of healthier coral reefs (Bruno et al., 2009; Done, 1992), Carlos Rosario was considered as a healthier coral reef than Palomino, and thus, presumably, one that has experienced less stressful environmental conditions than Palomino.

### Colony density, cover and size structure

To estimate the density and size structure of *O. annularis* and *P. astreoides* we haphazardly placed thirty 1 m^2^ quadrats along three 30 m belt transects on September 2012 at a depth of 6–8 m. When we placed the quadrats to compare colony size and density between both coral species we were basically measuring *P. astreoides* colonies and ramets from different genets of *O. annularis*. As Edmunds (2015) commented, live tissue of *O. annularis* is restricted to the upper surface of numerous lobes, each lobe forming a ramet showing self-sufficient areas of tissue irrespective of skeletal connection with other colonies. Therefore, we could compare measurements from both species. Quadrats were placed sequentially along the 30 m transects. So, all colonies of both species present were photographed. Photographs were analyzed sing the software Coral Point Count 4.1 software (Kohler & Gill, 2006) to measure the planar surface area using a “by the side” scale. Our scale was a 10 cm long PVC pipe that was placed by the side of each colony to calibrate the measurement. A two-way ANOVA was performed to determine whether colony size differs between species and localities. Sigma Stat 12 software was used to perform the calculations. Coral cover was estimated by overlying 100 points randomly over each of the 1 m^2^ quadrats and dividing the number of points over a coral by the total points superimpose.

### Life cycle transition rates

For the demographic analysis, we haphazardly selected at least 30 colonies of each of the three size categories of each species along the entire transect. This guaranteed a balanced and representative sample of the entire population size structure of the studied locality. A total of 100 colonies of each species were tagged and mapped in each site within an area of approximately 60 m^2^ (30 m long × 2 m width transect) at depths of 6–8 m with an annual tidal range of 0.57 m. Colonies were identified using numbered tags fixed with masonry nails to non-living substrate adjacent to the colony. Colony fate and growth was followed once a year from September 2012 to September 2014. All colonies with no recognizable living tissue or those that disappeared were considered dead. Also, in every census, we sampled thirty 1 m^2^ permanent quadrats randomly established within the study area to estimate recruitment rates of both species. Any new colony that was discernible with the naked eye was considered a coral recruit (see Irizarry-Soto & Weil (2009) for size estimates).

The demographic data were used to construct a size-based transition matrix (Caswell, 2001). The population of both species was divided into three size classes: 0–50 cm^2^ (Small or S), 50–150 cm^2^ (Medium or M) and >150 cm^2^ (Large or L) following Hernández-Pacheco, Hernández-Delgado & Sabat (2011). Equation (1) describes the matrix model in which the number of colonies in each of the size classes at time *t* + 1 equals to:

(1) $${\left( {\matrix{{{S}}\cr{{M}}\cr{{L}}\cr} } \right)_{\!\! t + 1}} = \left( {\matrix{ {P1} & {{R_{{\rm{SM}}}}} & {{R_{{\rm{SL}}}}}\cr{G1} & {P2} & {{R_{{\rm{ML}}}}}\cr0 & {G2} & {P3}\cr} } \right){\left( {\matrix{ {{S}}\cr{{M}}\cr{{L}}\cr} } \right)_{\!\! t}}$$

The contribution of each size class at time (*t*) to all others at (*t* + 1) is contained within the matrix that projects the population vector between *t* and *t* + 1 (see Eq. 1). *P*1, *P*2, and *P*3 stand for the probability of colonies of each size (S, M, and L, respectively) that survived and stayed in the same size class for one year (stasis). The *G*s represents the proportion of small and medium colonies to grow to the next size class. *R*s correspond to size retrogressions or shrinkage (i.e., the proportion of colonies that decreased in size due to tissue loss due to partial mortality).

Sexual recruitment was only considered in the matrices for *P. astreoides* because no recruits of *O. annularis* were found during the study period (2012–2014). To obtain the contribution of medium and large *P. astreoides* colonies to the small size class through sexual reproduction, we estimated the size-specific fecundity value by dividing the number of recruits for each site and census period by the number of medium and large-sized colonies. Only colonies over 70 cm^2^ were used for this calculation since this is the smallest reproductive size reported for *P. astreoides* (McGuire, 1998).

The matrices were used to calculate the asymptotic population growth rate (λ, the dominant eigenvalue of each matrix). To determine whether λs differ significantly between sites and species, transition matrices were subject to a bootstrapping re-sampling procedure (10,000 simulations) to obtain the 95% CI for λ (i.e., Mercado-Molina et al., 2015). A life table response experiment (LTRE) analysis was performed to determine the contribution of each transition to the observed differences in λ between sites and time periods (see Mercado-Molina et al., 2015). LTRE is a retrospective analysis that provides information on how much variation in a certain life cycle transition contributed to the observed differences in λ between treatments (years and localities in this study).

The transition probabilities were estimated by constructing transition frequency tables (Caswell, 2001) for each locality and census period (2012–2013 and 2013–2014). The three size classes were chosen to incorporate size-specific patterns of vital transition rates while maintaining a sample size greater than 25 colonies for each size category. Four transition matrices were generated, one for each site and census period.

To determine whether the transition probabilities in these four matrices were independent of time and location, log-linear models were applied to a four-way contingency table. Following Caswell (2001), fate (*F*) was set as the response variable and time (*T*), and location (*L*) were set as the explanatory factors, conditional upon initial colony size (*S*). The model that best fit the data was determined using the scaled Akaike information criteria (AIC), calculated for log-linear models as

(2) $${\rm{AIC}} = G2-2(df)$$

where *G*2 is the goodness-of-fit log-likelihood ratio statistic obtained from comparing the model with the saturated model and *df* is the degrees of freedom of the test (Caswell, 2001). Log-linear models were also applied to a three-way contingency table developed for each of the size classes to examine the effect of time and location on the fate of colonies within each size class. As suggested by Fingleton (1984), 0.5 was added to each cell value within the contingency tables to avoid estimation problems for values equal to 0. R.3.0.1 package popbio (Stubben & Milligan, 2007; R Development Core Team, 2014) was used to perform all demographic analyses. For log-linear analyses, the package MASS was used (Venables & Ripley, 2002).

## Results

### Density, cover and size structure

**Figure 2:**
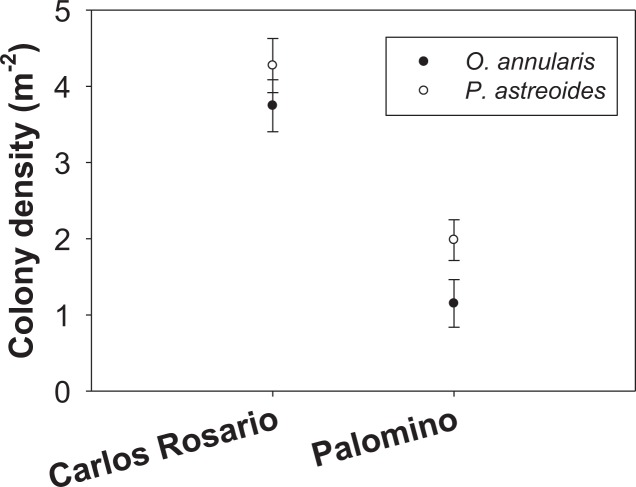
Colony density (m^−2^) for two coral species at both study sites.

The number of colonies per m^2^ of *P. astreoides* in Carlos Rosario (4.27 ± 0.36 SE) was significantly higher than for *O. annularis* (3.75 ± 0.34 SE) (Fig. 2). The same pattern was true in Palomino (*P. astreoides*: 1.98 ± 0.26 SE; *O. annularis*: 1.15 ± 0.31 SE; Fig. 2). However, no interaction was found between sites and species (Table 1). We did find significant differences when comparing the colony density (sites pooled) with *P. astreoides* showing higher densities of colonies (3.14 ± 0.24 SE) than *O. annularis* (2.47 ± 0.25 SE) (Table 1). We, also, found significant differences when comparing density within sites (species pooled) with Carlos Rosario showing higher values (4.01 ± 0.25 SE) than Palomino (1.57 ± 0.21 SE) (Table 1).

**Table 1:** Two-way ANOVA for the colony density analysis.

Source of variation	*df*	Sum squares	Mean square	*F*	*P*
Species	1	28.372	28.372	4.466	0.036
Site	1	365.525	365.525	57.542	<0.001
Species × site	1	1.425	1.425	0.224	0.636
Residual	241	1530.909	6.352		
Total	244	1925.363	7.891		

**Figure 3:**
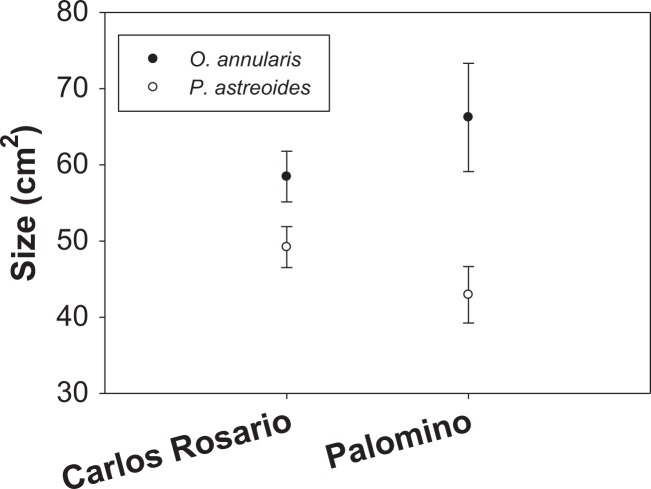
Mean colony size (cm^2^) structure for both species at each study site.

Mean colony size (cm^2^) of *O. annularis* in Carlos Rosario (58.47 ± 3.32 SE) was larger than *P. astreoides* (49.21 ± 2.70 SE) (Fig. 3). The same pattern was observed in Palomino, where mean colony size was 66.23 cm^2^ (±7.10 SE) and 42.94 cm^2^ (±3.70 SE) for *O. annularis* and *P. astreoides*, respectively (Fig. 3). Like the density data, no interaction was found between sites and size of species (Table 2) and no significant differences were found on mean colony size between sites (species pooled). We did find significant differences when comparing the size of species (sites pooled) with *O. annularis* showing larger colonies (62.35 ± 3.09 SE) than *P. astreoides* (46.08 ± 2.29 SE) (Table 2).

**Table 2:** Two-way ANOVA for the size distribution analysis.

Source of variation	*df*	Sum squares	Mean square	*F*	*P*
Species	1	38253.1	38253.1	11.096	<0.001
Site	1	81.632	81.632	0.0237	0.878
Species × site	1	7110.766	7110.766	2.063	0.151
Residual	1,022	3,523,277	3447.433		
Total	1,025	3,565,008	3478.056		

### Survival

No significant spatial differences were found when comparing survival rates of the two species between sites during the study period (2012–2014). At Carlos Rosario, 73% of *O. annularis* colonies survived, compared with 67% at Palomino (*X*^2^ = 1.28; *df* = 1; *P* > 0.05). *P. astreoides* colonies in Carlos Rosario showed a 73% survival rate compared to 78% in Palomino (*X*^2^ = 0.75; *df* = 1; *P* > 0.05). When comparing both species (sites and size classes pooled), *O. annularis* showed a lower survival rate (70%) compared to *P. astreoides* (75%), but the difference was not significant (*X*^2^ = 1.97; *df* = 1; *P* > 0.05).

### Growth, stasis, and retrogression

During the first census period (September 2012–September 2013) and pooling both sites, the most frequent transition for *O. annularis* was stasis (colonies remaining within their original size classes) of the small-sized colonies (31%). The growth of *O. annularis* was mainly from small to medium-sized colonies (7%). Regarding shrinkage, the highest transition found was medium to small-sized colonies (5%). In Carlos Rosario, the percentage of small- and medium-sized colonies of *O. annularis* that grew to a larger class (16%) was higher than that of medium- and large-sized colonies that shrank (9%). Contrastingly, in Palomino, more *O. annularis* colonies shrank (23%) as compared to colonies that grew to larger classes (11%). Like *O. annularis*, the most frequent transition for *P. astreoides* was stasis from small-sized colonies (40%), both sites pooled. Shrinking occurred mainly from medium to small-sized colonies (5%). *P. astreoides* grew mostly from medium to large-sized colonies (5%). *P. astreoides* in Carlos Rosario showed a similar pattern to *O. annularis* with a higher percentage of colonies growing (20%) compared to the ones that shrank (9%). However, in Palomino, 14% of *P. astreoides* colonies showed size progression, while 42% shrank.

For the second-year census period (September 2013–September 2014), the most frequent transition found for *O. annularis* was stasis in small-sized colonies (21%). The growth of *O. annularis* was mainly from medium to large-sized colonies (9%), and shrinkage, from large to medium-sized colonies (3%). Like the first census period, Carlos Rosario showed a higher percentage of *O. annularis* colonies (small-sized colonies + medium-sized colonies) that grew to a larger class (29%) than the colonies (medium-sized colonies + large-sized colonies) that shrank (7%). Contrastingly in Palomino, there were more *O. annularis* colonies that shrank (13%) than the ones that grew (9%).

Like *O. annularis*, for the second census and sites pooled, the most frequent transition found for *P. astreoides* was stasis from small-sized colonies (31%). Shrinking occurred manly from medium to small-sized colonies (9%). *P. astreoides* grew mostly from small to large-sized colonies (3%). *P. astreoides* in Carlos Rosario showed a higher percentage of colonies that shrank (29%) compared to the ones that grew (3%). In Palomino, the same pattern was observed, with 25% of the *P. astreoides* colonies exhibiting shrinkage and 11% growth.

### Population growth

During the first year of the study, populations of both species showed λ values below 1.0 (decreasing population). During the first year, populations of *O. annularis* exhibited λs below equilibrium at Carlos Rosario (0.817) and Palomino (0.694), followed by a decline at both sites during the second year (0.700 and 0.667, respectively). Populations of *P. astreoides* showed higher λs than *O. annularis* during the first census period at Carlos Rosario (0.898) and Palomino (0.894) with a decline at one of the sites (0.681 and 0.893) during the second census period. Significant differences (based on 95% CI) where observed when comparing populations of *O. annularis* between sites (Table 3). In contrast, λs did not vary considerably spatially for *P. astreoides* (Table 3). When comparing λs between species within sites *O. annularis* showed lower rates of population growth rates than *P. astreoides* at Palomino but not in Carlos Rosario. For the second year, λs decreased for both species and sites with a slight reduction found in Palomino, but no significant differences were found (based on 95% CI) (Table 3). *O. annularis* in C. Rosario showed a slightly higher λ value compared to Palomino but a lower λ value when compared to the first census. Contrastingly, *P. astreoides* in C. Rosario showed a notable decrease in λ than Palomino and compared to the first census.

**Table 3:** Size class transition probabilities of small-sized colonies (S: 0–50 cm^2^), medium-sized colonies (M: 51–150 cm^2^), large-sized (L: >150 cm^2^) and proportion of dead (D) colonies of *O. annularis* and *P. astreoides* at two localities (Carlos Rosario, Culebra and Palomino, Fajardo) in Eastern Puerto Rico during two annual time periods.

Size class	2012–2013				2013–2014		
	S	M	L		S	M	L
*O. annularis*							
C. Rosario							
S	0.673	0.066	0	S	0.363	0.066	0
M	0.163	0.666	0.142	M	0.090	0.166	0.090
L	0	0.166	0.500	L	0.151	0.333	0.636
D	0.163	0.100	0.357	D	0.393	0.433	0.272
λ	0.817 (95% CI 0.791–0.915)			λ	0.700 (95% CI 0.573–0.894)		
Palomino							
S	0.500	0.400	0	S	0.620	0.062	0.045
M	0.086	0.400	0.142	M	0	0.312	0.136
L	0.043	0.066	0.500	L	0.034	0.187	0.545
D	0.369	0.133	0.357	D	0.344	0.437	0.272
λ	0.694 (95% CI 0.686–0.825)			λ	0.667 (95% CI 0.636–0.883)		
*P. astreoides*							
C. Rosario							
S	0.608	0.275	0.200	S	0.551	0.429	0.150
M	0.065	0.437	0.230	M	0	0.363	0.230
L	0	0.187	0.769	L	0	0.090	0.615
D	0.326	0.187	0	D	0.448	0.181	0.153
λ	0.898 (95% CI 0.837–0.982)			λ	0.681 (95% CI 0.642–0.909)		
Palomino							
S	0.755	0.466	0.503	S	0.512	0.482	0.189
M	0.066	0.350	0.312	M	0.051	0.214	0.090
L	0.022	0.250	0.250	L	0.1025	0	0.818
D	0.155	0.100	0.187	D	0.333	0.428	0.090
λ	0.894 (95% CI 0.824–0.934)			λ	0.893 (95% CI 0.777–1.000)		

**Note:**

λ = estimated population growth rate; numbers within () represent the lower and upper 95% CI.

**Figure 4:**
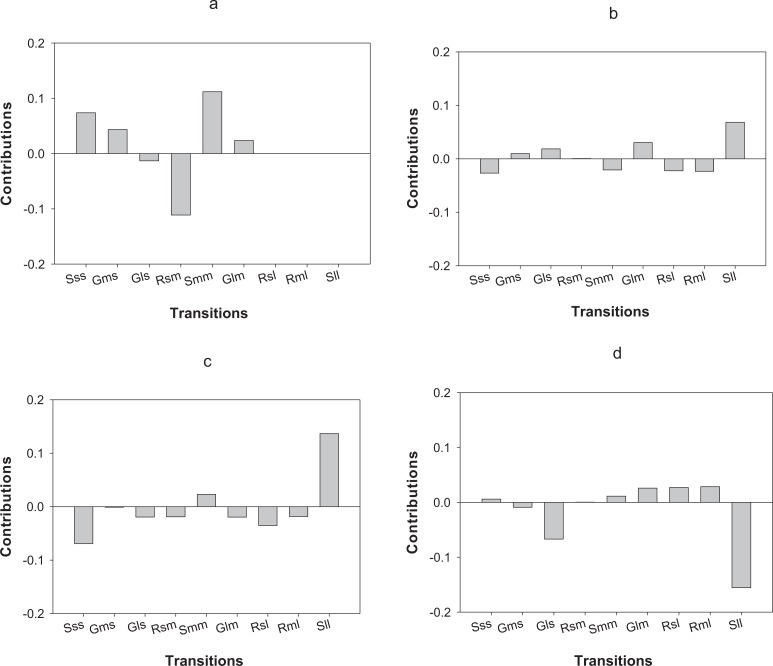
Results of the LTRE analysis showing the contribution of each life cycle transition to the population growth rate of *O. annularis* between Carlos Rosario and Palomino from (A) 2012–2013 and from (B) 2013–2014. LTRE analysis of *P. astreoides* between Carlos Rosario and Palomino from (C) 2012–2013 and from (D) 2013–2014 is also shown. Positive and negative values indicate transitions that contribute to and suppress local population growth, respectively. S, stasis; G, growth; R, retrogression; s, small; m, medium; l, large.

LTRE analysis indicates that differences in the first year of the study in *O. annularis* growth rates between sites were mainly due to a lower proportion of retrogression (i.e., colonies that decreased in size) from medium to small-sized colonies (Rsm) and higher values of stasis (i.e., colonies that stayed in the same size class) of medium-sized (Smm) colonies (Fig. 4A) found in Carlos Rosario (Table 3). In the second year (2013–2014), differences were mainly due to the higher proportion of stasis of large-sized colonies (Sll; Fig. 4B) found in Carlos Rosario (Table 3).

Differences in λs observed in *P. astreoides* between Carlos Rosario and Palomino during the first year of study were mainly due to a high proportion of stasis of large-sized colonies (Sll; Fig. 4C) found in Carlos Rosario (Table 3). However, in the second year of the study, the reduction in λ for *P. astreoides* in Carlos Rosario was mainly caused by a decrease in the proportion of stasis of large-sized colonies (Sll; Fig. 4D).

**Figure 5:**
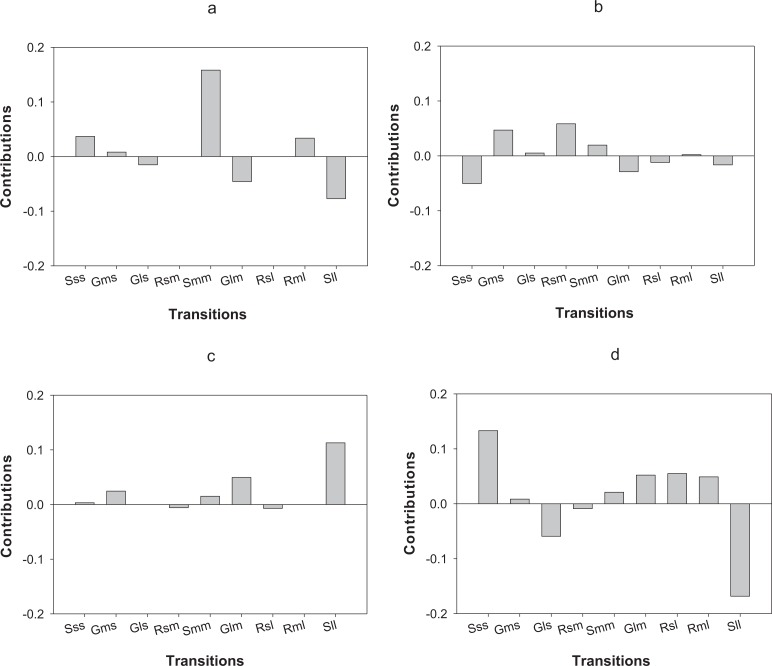
Results of the LTRE analysis showing the contribution of each life cycle transition to the population growth rate of *O. annularis* between the two census periods (2012–2013 and 2013–2014) in (A) Carlos Rosario and in (B) Palomino. LTRE analysis of *P. astreoides* between the two census periods in (C) Carlos Rosario and in (D) Palomino is also shown. Positive and negative values indicate transitions that contribute to and suppress local population growth, respectively. S, stasis; G, growth; R, retrogression; s, small; m, medium; l, large.

In terms of differences in λs between census periods, populations of *O. annularis* in Carlos Rosario showed a decrease from 0.80 to 0.70 (Table 3). The higher growth rates experienced by the populations during 2012–2013 was most probably due to a higher survival rate of medium-sized colonies that remained in the same size category (Fig. 5A). The decrease in λs between census periods found in *P. astreoides* populations in Carlos Rosario from 0.89 to 0.68 (Table 3) was mainly due to a higher survival rate of large-sized colonies that remained in the same size category during the first year (Fig. 5C).

### Recruitment

No recruits were found for *O. annularis* during the study period (2012–2014). In contrast, we found a higher number of *P. astreoides* recruits m^−2^ y^−1^ (mean 0.62 ± 0.09 SE) in Palomino compared to Carlos Rosario (mean 0.35 ± 0.08 SE) during the study period.

### Log-linear analysis

The results of the four-way contingency table indicate that locality (*L*) had a significant effect on the demographic rates of both species (Tables S1 and S2). When comparing each model to the saturated model (TLSF), the model that considered the effects of location while excluding the effect of time (TLS, FSL) was the one with the best goodness of fit (Tables S1 and S2). That is, variability among size class transitions are better explained by the independent effect of location than by the interaction between location and time. Nevertheless, the model that included the effect of location and time simultaneously (TLS, FST, FSL) had comparable AIC values to that of the model TLS, FSL (Tables S3 and S4); therefore, TLS, FST, FSL could also be considered a good approximation of the observed spatiotemporal variation. The ΔAIC values for TLS, FST, FSL were 2.28 and 2.26 for *O. annularis* and *P. astreoides*, respectively, indicating that this model has better support than the other models (Tables S3 and S4).

## Discussion

The objectives of this study included a comparison of the vital rates of two common scleractinians in Caribbean coral reefs that are thought to be stress-resistant (Darling et al., 2012): *O. annularis* and *P. astreoides*. In general, we found that for the studied populations (1) stasis of small-sized colonies was the life cycle transition that contributes the most to local population growth rates; (2) colony shrinkage was more common than colony growth; (3) colony fate was more dependent on spatial rather than on time variability; and (4) population growth rates were below equilibrium.

In comparisons of size progression (e.g., growth) and shrinkage (e.g., size reduction) of colonies during the whole census period, both species showed higher rates of size retrogression, particularly in Palomino, the location classified as degraded. These results are consistent with previous studies concluding that partial mortality is more prevalent in reefs with “suboptimal” environmental conditions (Nugues & Roberts, 2003; Wielgus et al., 2010; Dikou & van Woesik, 2006). The dominance of small colonies (positive skewing) within coral populations is a common response to environmental stress episodes (Hughes & Tanner, 2000; Edmunds & Elahi, 2007). Positive skewing found on *O. annularis* may be due to episodes such as disease, physiological stress, or algal overgrowth that reduce colony size by partial mortality or fission (Edmunds & Elahi, 2007). Demographic analysis and modeling have indicated that the vital rates of *O. annularis* are very sensitive to bleaching, and that interval of major events of less than 17 years will result in population reduction that will seriously compromise the viability of *O. annularis* populations (Hernández-Pacheco, Hernández-Delgado & Sabat, 2011). However, in our two-year census study, no major disturbances such as hurricanes, bleaching, predator outbreaks, or epizootic events directly impacted the two study sites. The only perturbing event during the study period was a heavy rainfall event (356 cm, an anomaly of 215% in relation to mean monthly rainfall) during June 2013 (Hernández-Delgado et al., 2014), three months before the first survey. Low salinity may weaken the coral physiological performance (Kerswell & Jones, 2003) with conceivable costs to colony growth. Therefore, our study suggests that even in the absence of a major disturbance, the vital rates of *P. astreoides* and *O. annularis* can be susceptible to minor or moderate local variations in environmental parameters. This pattern concurs with a previous study about the population vital rates of *Acropora cervicornis* within the same localities (Mercado-Molina et al., 2015). Even with these environmental conditions, λs in both sites were <1 perhaps due to the zero-recruitment found for *O. annularis*.

Results of the log-linear analysis for both species suggest that location is a better predictor of colony fate than time. Mercado-Molina et al. (2015), working in the same area, found comparable results for the threatened coral *A. cervicornis*. In the current study, the log-linear analysis showed that large-sized colonies of *O. annularis* were not sensitive to the effect of location or time. This contrast with previous studies where the largest effect of *O. annularis* population decline have been attributed to the mortality of large colonies (Hughes & Tanner, 2000; Edmunds & Elahi, 2007). On the other hand, in *P. astreoides* none of the size classes was clearly vulnerable to spatio-temporal variability. However, it was observed that both small and large colonies had a high probability to survive and remain within their size class. This observation coincides with a previous demographic study showing that stasis is the most common demographic transition in *P. astreoides* (Edmunds, 2010).

The observed spatio-temporal similarity in the pattern of demographic transitions can lead us to conclude that both species behave in similar ways from the perspective of their demography, which is consistent with our hypothesis, that if *O. annularis* is a stress-tolerant species, then its demography would be comparable to that of a stress-tolerant species, *P. astreoides*. Nevertheless, to be considered demographically resilient, a population should exhibit low spatial and temporal variability of its vital rates. For instance, both species showed considerable spatio-temporal variability in rates of demographic transitions as well as in λ values. The drastic decline in population growth rates experienced by the two species at Carlos Rosario was somehow surprisingly, not only because it occurred during a period with no major disturbances but also because the site seems to have appropriate environmental conditions for coral development (e.g., clear water, low algal cover). Interestingly, Mercado-Molina et al. (2015) also documented a considerable reduction in population growth rates in the coral *A. cervicornis* at Luis Peña reef, which is adjacent and has similar environmental conditions to Carlos Rosario. A possible explanation is that corals at Carlos Rosario were more susceptible to even slight changes in the prevailing environmental conditions, i.e., factors that are not necessarily considered major disturbances, such as an increase in heavy rainfall (as mentioned above). Corals that have survived poor conditions tend to be stronger immunologically and may be adapted to high environmental variability (Baker et al., 2004; Baker, Glynn & Riegl, 2008).

After the mass bleaching in 2005, *O. annularis* colonies within the Carlos Rosario site suffered a population decline but recovered three years after the event (Hernández-Pacheco, Hernández-Delgado & Sabat, 2011). A similar pattern of recovery was found in Tektite Reef in St. Johns (Edmunds, 2015). Edmunds (2015) proposed that *O. annularis* shows ecological resilience because of the increased coral cover within the Tektite Reef and projected a continued population growth in years to come if no disturbance impacts this site. He concluded that this resilience was found mainly because of the deep habitat (14 m) in which these colonies are found. At this depth colonies would be alleviated from the effects of elevated temperature bleaching due to lower light intensity. Contrastingly in our study, *O. annularis* populations showed a decrease in growth even with no major environmental conditions affecting these colonies. However, our study did not include a deep transect and all colonies sampled were at depths shallower than 8 m.

We found the lower intrinsic rate of population growth (λ) for *P. astreoides* colonies (0.68–0.89) rates than previously reported for other Caribbean reefs (0.95–1.02) (Edmunds, 2010). However, similar λ values (0.89) have been reported in restored coral reef sites in Florida (Lirman & Miller, 2003). Low and decreasing population growth in *P. astreoides* was unexpected since an increase in abundance of these colonies has been projected over the next 100 years even with recurrent disturbance events and variable recruitment (Green, Edmunds & Carpenter, 2008; Edmunds, 2010). Conversely, we found *O. annularis* colonies showing λs <1 (0.69–0.81) during the two years this study was performed. Similar values have been found for these colonies (0.58–0.67) in other Caribbean reefs (Edmunds & Elahi, 2007).

Population growth of *P. astreoides* was 0.89 for both sites in the first year and for Palomino in the second year. In the second year, the population in Carlos Rosario showed λ of 0.68, the lowest for these colonies. If we compare the recruitment rates of this species within both sites, colonies in Carlos Rosario showed a significant lower recruitment rate. Perhaps the lower λ values found on this site is because population growth of *P. astreoides* is susceptible to variations in recruitment rates (Lirman & Miller, 2003). Recruitment rates in Carlos Rosario are similar to what Lirman & Miller (2003) found in Florida (0.30 m^2^ y^−1^). Such recruitment rates are considered moderate for this species. The recruitment rate found in Palomino (0.62 m^2^ y^−1^) may be considered as high for *P. astreoides* which may result in an increase in population abundance in the near future (Lirman & Miller, 2003). However, λs of *P. astreoides* were below 1 even with the high recruitment in Palomino. Previous studies have shown even higher recruitment rates for these colonies (0.98–1.64 individuals 0.25 m^−2^) in St Johns (Edmunds, 2010). Perhaps the population growth below equilibrium found in the current study for *P. astreoides* is because of highly variable recruitment rates (Lirman & Miller, 2003).

The comparable variability in λs found in our study within both sites suggests that “weedy” (*P. astreoides*) and “stress tolerant” (*O. annularis*) (Darling et al., 2012) corals may behave similarly in terms of their demographics under a scenario of no major stressful event. The higher recruitment rates for *P. astreoides* found in the “degraded” Palomino may support other studies highlighting the ability of this species to exploit areas that are normally not suitable for planulae settlement (in terms of macroalgal cover). Similar survival rates of both species within both sites suggest comparable demographic resilience of both coral species in a “degraded” and “healthy” site and that *O. annularis* may survive in stressful environments (in terms of macroalgal cover) such as Palomino. Perhaps the *O. annularis* colonies in this low coral/high macroalgal cover conditions have invested more energy into survival and have adapted to these stressful conditions.

In terms of coral reef management, we propose that coral colonies surviving, but most important recruiting, in sites with high macroalgal/low coral cover (such as Palomino) are an important aspect of future resiliency in coral reef environments. Palomino site is one of the most visited sites by recreational vessels within the Eastern part of Puerto Rico and is used by a major hotel which takes its clients to visit the island as well (Shivlani, 2009) showing high anthropogenic impacts in its marine ecosystems (Hernández-Delgado et al., 2012). Carlos Rosario reef, the high coral/low macroalgal cover site, is located within a marine protected area and 30 km apart from the main island of Puerto Rico. However, we observed similar patterns of vital rates and population growth rates of both species within both locations and even higher recruitment of *P. astreoides* in Palomino. These findings concur with the recent theory that locally impacted reefs might be less sensitive to global stressors than remote, more pristine, reefs (Bruno & Valdivia, 2016; Coté & Darling, 2010) because corals that survive in impacted reefs may be more resistant to climate change effects. Bruno & Valdivia (2016) found no correlation between coral/macroalgal cover with isolation from local anthropogenic stressors around the world. We need to look at the coral population dynamics within the “degraded” coral reefs and start asking questions to understand better how to deal with the issue of promoting resilience in the coral reef environment (Coté & Darling, 2010).

## Supplemental Information

10.7717/peerj.3906/supp-1 Supplemental Information 1 Supplemental Table 1: Log-linear analysis of the effect of location and time on fate of *Orbicella annularis* colonies of the three size classes. *F* = Fate, *S* = Stage, *T* = Time, *L* = Location, ^a^ = *p* < 0.05, ^b^*p* > 0.05, G-squared = goodness of fit, *df* = degrees of freedom.

10.7717/peerj.3906/supp-2 Supplemental Information 2 Supplemental Table 2: Log-linear analysis of the effect of location and time on fate of *Porites astreoides* colonies of the three size classes. *F* = Fate, *S* = Stage, *T* = Time, *L* = Location, ^a^ = *p* < 0.05, ^b^*p* > 0.05, G-squared = goodness of fit, *df* = degrees of freedom.

10.7717/peerj.3906/supp-3 Supplemental Information 3 Supplemental Table 3: AIC values for each of the models applied to the transition data obtained for *Orbicella annularis*. *F* = Fate, *S* = Stage, *T* = Time, *L* = Location, G-squared = goodness of fit, *df* = degree of freedom.

10.7717/peerj.3906/supp-4 Supplemental Information 4 Supplemental Table 4: AIC values for each of the models applied to the transition data obtained for *Porites astreoides*. *F* = Fate, *S* = Stage, *T* = Time, *L* = Location, G-squared = goodness of fit, df = degree of freedom.

10.7717/peerj.3906/supp-5 Supplemental Information 5 Raw data.

## Additional Information and Declarations

### Data Availability

The following information was supplied regarding data availability: The raw dataset has been provided as Supplemental Dataset Files.
