# Supplementary material for: Comparative demography of two common scleractinian corals: Orbicella annularis and Porites astreoides
Source: PeerJ. 2017 Oct 27;5:e3906. doi: 10.7717/peerj.3906 (PMC5661470; doi:10.7717/peerj.3906)
Supplement: Supplemental Information 1 — F = Fate, S = Stage, T = Time, L = Location, a = p < 0.05, bp > 0.05, G-squared = goodness of fit, df = degrees of freedom. [file peerj-05-3906-s001.docx]

|  |  | G ^2^ | | |  |  | | G ^2^ | |
| --- | --- | --- | --- | --- | --- | --- | --- | --- | --- |
| 3-way Model | df | Small | Medium | Large |  | 4-way model | df | | Sum |
| 1. TL, F | 9 | 21.71 | 29.19 | 1.42 |  | TLS,FS | 41 | | 241.69 |
| 2. TL, FL | 6 | 13.82 | 9.07 | 0.34 |  | TLS,FSL | 38 | | 220.73 |
| Effect of location | 3 | 7.89^a^ | 20.12^a^ | 1.08^b^ |  |  | 3 | | 20.96^a^ |
|  |  |  |  |  |  |  |  | |  |
| 1. TL, F | 9 | 21.71 | 29.19 | 1.42 |  | TLS,FS | 41 | | 241.69 |
| 3. TL, FT | 6 | 17.73 | 23.94 | 1.38 |  | TLS,FST | 38 | | 238.47 |
| Effect of time | 3 | 3.98^b^ | 5.25^b^ | 0.04^b^ |  |  | 3 | | 3.22^b^ |
|  |  |  |  |  |  |  |  | |  |
| 3. TL, FT | 6 | 17.73 | 23.94 | 1.38 |  | TLS,FST | 38 | | 238.47 |
| 4. TL, FT, FL | 3 | 9.72 | 3.63 | 0.29 |  | TLS,FST,FSL | 35 | | 217.01 |
| Effect of location  (given the effect of time) | 3 | 8.01^a^ | 20.31^a^ | 1.09^b^ |  |  | 3 | | 21.46^a^ |
|  |  |  |  |  |  |  |  | |  |
| 2. TL, FL | 6 | 13.82 | 9.07 | 0.34 |  | TLS,FSL | 38 | | 220.73 |
| 4. TL, FT, FL | 3 | 9.72 | 3.63 | 0.29 |  | TLS,FST,FSL | 35 | | 217.01 |
| Effect of time  (given the effect of location) | 3 | 4.1^b^ | 5.44^b^ | 0.05^b^ |  |  | 3 | | 3.72^b^ |
|  |  |  |  |  |  |  |  | |  |
| 4. TL, FT, FL | 3 | 9.72 | 3.63 | 0.29 |  | TLS,FST,FSL | 35 | | 217.01 |
| 5. TLF | 0 | 0 | 0 | 0 |  | TLSF | 0 | | 0 |
|  | 3 | 9.72^a^ | 3.63^b^ | 0.29^b^ |  |  | 35 | | 217.01^a^ |

**Supplemental file Table 1: Log-linear analysis of the effect of location and time on fate of *Orbicella annularis* colonies of the three size classes. F= Fate, S=Stage, T=Time, L=Location, ^a^=p<0.05, ^b^p>0.05, G-squared = goodness of fit, df= degrees of freedom.**
