# Supplementary material for: Comparative demography of two common scleractinian corals: Orbicella annularis and Porites astreoides
Source: PeerJ. 2017 Oct 27;5:e3906. doi: 10.7717/peerj.3906 (PMC5661470; doi:10.7717/peerj.3906)
Supplement: Supplemental Information 2 — F = Fate, S = Stage, T = Time, L = Location, a = p < 0.05, bp > 0.05, G-squared = goodness of fit, df = degrees of freedom. [file peerj-05-3906-s002.docx]

|  |  | G ^2^ | | |  |  | | G ^2^ | |
| --- | --- | --- | --- | --- | --- | --- | --- | --- | --- |
| 3-way Model | df | Small | Medium | Large |  | 4-way model | df | | Sum |
| 1. TL, F | 9 | 15.05 | 9.23 | 13.77 |  | TLS,FS | 41 | | 220.67 |
| 2. TL, FL | 6 | 8.10 | 3.40 | 9.84 |  | TLS,FSL | 38 | | 211.12 |
| Effect of location | 3 | 6.95^b^ | 5.83^b^ | 3.93^b^ |  |  | 3 | | 9.55^a^ |
|  |  |  |  |  |  |  |  | |  |
| 1. TL, F | 9 | 15.05 | 9.23 | 13.77 |  | TLS,FS | 41 | | 220.67 |
| 3. TL, FT | 6 | 8.89 | 8.40 | 9.61 |  | TLS,FST | 38 | | 217.49 |
| Effect of time | 3 | 6.16^b^ | 0.83^b^ | 4.16^b^ |  |  | 3 | | 3.18^b^ |
|  |  |  |  |  |  |  |  | |  |
| 3. TL, FT | 6 | 8.89 | 8.40 | 9.61 |  | TLS,FST | 38 | | 217.49 |
| 4. TL, FT, FL | 3 | 1.75 | 2.54 | 6.01 |  | TLS,FST,FSL | 35 | | 207.38 |
| Effect of location  (given the effect of time) | 3 | 7.14^b^ | 5.86^b^ | 3.60^b^ |  |  | 3 | | 10.11^a^ |
|  |  |  |  |  |  |  |  | |  |
| 2. TL, FL | 6 | 8.10 | 3.40 | 9.84 |  | TLS,FSL | 38 | | 211.12 |
| 4. TL, FT, FL | 3 | 1.75 | 2.54 | 6.01 |  | TLS,FST,FSL | 35 | | 207.38 |
| Effect of time  (given the effect of location) | 3 | 6.35^b^ | 0.86^b^ | 3.83^b^ |  |  | 3 | | 3.74^b^ |
|  |  |  |  |  |  |  |  | |  |
| 4. TL, FT, FL | 3 | 1.75 | 2.54 | 6.01 |  | TLS,FST,FSL | 35 | | 217.01 |
| 5. TLF | 0 | 0 | 0 | 0 |  | TLSF | 0 | | 0 |
|  | 3 | 1.75^b^ | 2.54^b^ | 6.01^b^ |  |  | 35 | | 217.01^a^ |

**Supplemental file Table 2: Log-linear analysis of the effect of location and time on fate of *Porites astreoides* colonies of the three size classes. F= Fate, S=Stage, T=Time, L=Location, ^a^=p<0.05, ^b^p>0.05, G-squared = goodness of fit, df= degrees of freedom.**
