# Supplementary material for: Comparative demography of two common scleractinian corals: Orbicella annularis and Porites astreoides
Source: PeerJ. 2017 Oct 27;5:e3906. doi: 10.7717/peerj.3906 (PMC5661470; doi:10.7717/peerj.3906)
Supplement: Supplemental Information 3 — F = Fate, S = Stage, T = Time, L = Location, G-squared = goodness of fit, df = degree of freedom. [file peerj-05-3906-s003.docx]

| Model | G^2^ | df | P | AIC | ΔAIC |
| --- | --- | --- | --- | --- | --- |
| 1. TLS, FS | 241.69 | 41 | 0.00 | 159.69 | 14.96 |
| 2. TLS, FST | 238.47 | 38 | 0.00 | 162.47 | 17.74 |
| 3. TLS, FSL | 220.73 | 38 | 0.00 | 144.73 | 0.00 |
| 4. TLS, FST, FSL | 217.01 | 35 | 0.00 | 147.01 | 2.28 |
| 5. TLSF | 0.00 | 0 | 1.00 | 0.00 | 144.73 |

**Supplemental file Table 3: AIC values for each of the models applied to the transition data obtained for *Orbicella annularis.* F= Fate, S=Stage, T=Time, L=Location. G-squared = goodness of fit. df= degree of freedom.**
