# Supplementary material for: Comparative demography of two common scleractinian corals: Orbicella annularis and Porites astreoides
Source: PeerJ. 2017 Oct 27;5:e3906. doi: 10.7717/peerj.3906 (PMC5661470; doi:10.7717/peerj.3906)
Supplement: Supplemental Information 4 — F = Fate, S = Stage, T = Time, L = Location, G-squared = goodness of fit, df = degree of freedom. [file peerj-05-3906-s004.docx]

| Model | G^2^ | df | P | AIC | ΔAIC |
| --- | --- | --- | --- | --- | --- |
| 1. TLS, FS | 220.67 | 41 | 0.00 | 138.67 | 3.55 |
| 2. TLS, FST | 217.49 | 38 | 0.00 | 141.49 | 6.37 |
| 3. TLS, FSL | 211.12 | 38 | 0.00 | 135.12 | 0.00 |
| 4. TLS, FST, FSL | 207.38 | 35 | 0.00 | 137.38 | 2.26 |
| 5. TLSF | 0 | 0 | 1 | 0 | 135.12 |

**Supplemental file Table 4: AIC values for each of the models applied to the transition data obtained for *Porites astreoides.* F= Fate, S=Stage, T=Time, L=Location. G-squared = goodness of fit. df= degree of freedom.**
